# Editorial Comment from Dr Isotani to Removal of an encrusted ureteral stent by cutting the stent with a holmium laser using 4.5‐Fr semi‐rigid and flexible ureteroscopes

**DOI:** 10.1002/iju5.12204

**Published:** 2020-08-01

**Authors:** Shuji Isotani

**Affiliations:** ^1^ Department of Urology Graduate School of Medicine Juntendo University Tokyo Japan

Stent encrustation constitutes one of the most difficult complications of ureteral stents. In this case report, Imai *et al.* presented the case of the encrusted ureteral stent that was successfully treated with an endoscopic procedure using retrograde approach.^1^ The knowledge about this case is important for the endocrinologists to understand the endoscopic treatment option for the encrusted ureteral stent.

As the authors described in this report, various management strategies for encrusted stents were reported, including open surgery, laparoscopic procedure, percutaneous nephrolithotomy, the endurological approach, and extracorporeal shock wave lithotripsy. Most encrusted and retained ureteral stents can be removed using endoscopic techniques. This minimally invasive approach is recommended as first‐line therapy. For the complete encrustation stent in the renal pelvis, the percutaneous nephrolithotomy approach is recommended. In their report, the authors reported their successful retrieval of the encrusted ureteral stents endoscopically by cutting twice it into three parts with a Ho:YAG laser using 4.5/6.5‐Fr semi‐rigid and flexible ureteroscope. Similar to this report, for the retaining encrusted ureteral stents, the endurological approach was reported more than before with developments of surgical devices including new endoscopy, laser technology, and accessories. The endurological approach can be a less invasive approach than others such as open, laparoscopic surgery, or percutaneous nephrolithotomy. They demonstrated very well their detailed method in the report. Some other modification of endurological methods were reported. In one report, the distal end of encrusted ureteral stents was straightening in the bladder without and pull it out of the urethra, then crush calculi around the stent by the ureteroscope with laser, then pull out the double‐J stent and remove whole double‐J stent as one.^2^ Another report demonstrated that they successfully removed encrusted ureteral stents by retrograde ureteroscopy and percutaneous nephrolithotripsy.^3^

It is important to know that now we have some options to manage encrustation stent, specially in minimum invasive endurological approach using a combination of ureteroscope and laser. We need to know these updated management methods including its limitation and consider what is the best clinical practice in our institution for encrustation stent. It may provide a real benefit for the patients.

Of course, we need to think it again, the best treatment is the prevention of this complication by providing detailed patient education and the development of a tracking system.

## Conflict of interest

The author declares no conflict of interest.
